# Supplementary material for: Herbarium-based studies on taxonomy, biogeography and ecology of Psilochilus (Orchidaceae)
Source: PeerJ. 2016 Nov 8;4:e2600. doi: 10.7717/peerj.2600 (PMC5103833; doi:10.7717/peerj.2600)
Supplement: Supplemental Information 1 [file peerj-04-2600-s001.doc]

**Annex 1.** Complete list of examined specimens.

Cuba. Oriente, slopes of La Bayamesa, crest of the Sierra Maestra near Aserradero San Antonio de los Cumbres, 1500-1800 m, 21-24 Jan 1956, *C. V. Morton 9226* (US), Oriente, crest of Sierra Maestra between Pico Turquino and La Bayamesa, 1350 m, 27-28 Oct 1941, *C. V. Morton, J. Acuna 3526* (US), Sierra Nipe, near Woodfred, oriente deciduoud woods and thickets, 450-550 m, 11 Dec 1909, *J. A. Shafer 3150* (NY), Loma Gardero S. Maeitro, 1 Aug 1935*, J. T. Roig, G. C. Bucher 6670* (NY), *Sine loc.*, 1856-1857, *C. Wright 615* (K, W). Dominica. St. Paul, trail leading to Morne Trois Pitons, 2500 ft., 14 Jun 1967, *D. C. Wasshausen, E. S. Ayensu 389* (US), Syndicate, Nortwhest slopes of Morne Diablotins. Near Picard gorge, 2 Oct 1984, *C. Whitefoord 4313* (BM), St. Peter. Near Picard gorge, Mar-Apr 1996, *C. Whitefoord 7358* (BM), *Sine loc*., *F. E. Lloyd 909* (NY). Dominican Republic. "Las Abejas", wet wooded valley about 10 miles W from Aceitillar, 1200-1300 m, 24 Feb 1969, *A. H. Liogier 14199* (NY, US), Firme de Banilejo, Piedra Blanca. Herbácea erguida o decumbente, de hasta 25 cm de alto, 800 m, 9 Aug 1973, *A. H. Liogier 19951* (NY), Prov. Santiago, District of San José de Las Matas, Arroyo Jiconié, 750 m, 8 Oct 1930, *E. J. Valeur 723* (K, MO, NY, US), Terrestre, en lugar húmedo y somrio sobrío la cima del Pico Igua, 960 m, 15 Aug 1946, *J. de J.S. Jimenez 1240* (US), Prov. Barahona, 1400 m, Apr 1912, *M. Fuertes 1483* (NY), Cachete, 20 May 2004, *P. Acevedo-Rdgz. et al. 13843* (NY, US), Sierra de Baoruco, prov. Barahona, Loma "Pie Pol" de La Guasára de Barahona, 1250 m, 25 Mar 1987, *T. Zanoni et al. 38622* (NY). Grenada. *Sine loc*., 1890-1891, *R. V. Sherring s.n.* (K). Guadeloupe. Malouba, 600-1000 m, 1893, *A. Duss 3344* (NY, US), Bains-Janis, 700 m, 13 Sep 1944, *A. Questel 4140* (US x 2), Soufrière, 1100 m, 10 Oct 1936, *H. Stehlé 1138* (NY), Dugomier (Ténèbe), 800 m, 5 Dec 1936, *H. Stehlé 1304* (NY), Bains Chauds du Matouba, 1050 m, 2 Jan 1937, *H. Stehlé 1381* (NY), Bains Chauds du Matouba, 1050 m, 2 Jan 1937, *H. Stehlé 2571* (NY), *Sine loc*., 1893, *A. Duss 3388* (NY), *Sine loc*., *A. Duss 3483* (NY). Haiti. Massif de la Hotte, western group, ca. 900 m, 27 Aug 1927, *E. L. Ekman 9000* (US), Gros Cheval, Morne des Commissares, 1600 m, 13 Sep 1944, *L. R. Holdridge 1951* (NY), Montagnes de la Hotte, 24 Aug 1927, *W. J. Eyerdam 348* (NY, US). Jamaica. St. Andrew, Mt. Horeb peak. Upper montane rainforest, 450 ft., 1 Feb 1977, *A. C. Podzorski JA12* (K), Portland, Abraham's Ridge, in woodland, beside trail in litter, 3000 ft., 17 Dec 1973, *B. D. Morley & C. Whitefoord 604* (BM), Below Vinegar Hill, 25 Aug 1896, *Botanical Departent 6462* (NY), Mabeco River, 2000 ft., 25 Jul 1903, *G. E. Nichols 160* (MO, NY, US), Ridge below Vinegar Hills, 3800 ft., 5 Feb 1908, *W. Harris 10097* (NY), Vinegar Hill, 4000-5000 ft., 25 Jun 1896, *W. Harris 6252* (K), Near Mabess River, 3000 ft., 23 Apr 1903, *W. R. Maxon 1530* (US), Southeastern slopes of Stone Joie Bump, St. Thomas, 600-800 m, 9 Jun 1926, *W. R. Maxon 9015* (NY, US), Upper southern slopes and summit of Maccasucker Bump, St. Thomas, 825-1025 m, 25 Jun 1926, *W. R. Maxon 9551* (US), *Sine loc*., *D. Morris 2090* (NY, W), *Sine loc*., 9 Feb 1903, *L. M. Underwood 1349* (NY). Martinique. Camp Colson, *A. Duss 4484* (NY, US). Puerto Rico. Utuado, Bo. Caonillas Ariba, Cerro Morales, upper NW slopes near summit area, 925-950 m, 19 Sep 1993, *F. Axelrod, P. Bayman 7038* (NY), Maricao, 19 Apr 1933, *F. H. Sargent 719* (US), Prope Adjuntas, 3 May 1886, *I. Urban 4246* (K), Prope Utuado, 7 Feb 1886, *I. Urban 4479* (W), Prope Utuado, in sylva primaria, 16 Mar 1887, *I. Urban 493* (NY), Mun. Maricao, Maricao Forest Reserve, western end of Las Tetas de Cerro Gordo above Rd 120, 830 m, 30 Jun 1989, *J. D. Ackerman 2619* (MO, US), Monte Alegrillo, 900 m, 3 Apr 1913, *N. L. Britton et al. 2603* (NY), Indiera Fria, near Maricao, 430-800 m, 19-22 Feb 1915, *N. L. Britton et al. 4461* (NY). Saint Vincent and the Grenadines. St. Vincent, 2200 ft., 1890, *H. H. Smith, G. W. Smith 1924* (K). Santo Domingo. Cordillera Central, prov. de Azusa, San Juán, Loma La Vieja, ca. 900 m, 28 Aug 1929, *E. L. Ekman 13422* (NY, US), Prope Conostanza, in sylvis umbrosis humidis, 1250 m, Aug 1910, *H. von Türckheim 3134* (BM, NY, W). Mexico. Chiapas, Finca El Suspiro, near Berriozabal, 25 Sep 1957, *R. L. Dressler 2257* (US). Nicaragua. Dept. Granada, summit of extinct volcano, Mombacho, 16 Dec 1973, *J. T. Atwood, D. A. Neill 6737* (MO), summit of extinct volcano, Mombacho, 20 Jan 1974, *J. T. Atwood, D. A. Neill 7057* (MO), Dept. Jinotega, Municipio de Wiwili, Reserva Cerro Kilambé, 1400-1750 m, 7 Sep 2000, *R. Rueda et al. 14958* (MO), Reserva Natural Kilambé, municipio de Bocay, Comunidad Santa Teresa de Kilambé, 900-1100 m, 8 Jan 2001, *R. Rueda et al. 15550* (MO), Dept. Matagalpa, Macizos de Peñas Blancas, SE side, drainage of Quebrada El Quebradon, peak WNW of Hda. San Martín, 1400-1600 m, 20 Jan 1982, *W. D. Stevens et al. 21228* (MO). Belize. Cayo, In forest along ridge, near ridge top camp in vicinity of Doyle's Delight. Southern Maya Mountains, 1098 m, 8 Dec 1993, *B. Allen 15276* (MO), Toledo, Camp 3, 6-8 km southeast of union Camp. Trail from camp 3 toward Cabro on the Jimmy cut trail, 665-700 m, 17 Feb 1997, *T. Hawkins 1463* (MO). Guatemala. Alta Verapaz, 5000 ft., Jan 1878, *H. von Türckheim 52* (W), 3800 ft., Sep 1886, *H. von Türckheim 789* (US), 1600 m, Dec 1907, *H. von Türckheim II1998* (US), Mountains along road between Tactic and the divide on road to Tamahú, 1500-1600 m, 1-7 Apr 1941, *P. C. Standley 91359* (F), Dept. San Marcos, above Finca El Porvenir, up to Loma Bandera Shac, lower south-facing slopes of Volcán Tajumulco, 1300-1500 m, 9 Mar 1940, *J. A. Steyermark 37344* (F), Dept. Huehuetenango, vicinity of Maxbal, about 17 miles north of Barillas, Sierra de los Cuchumatanes, 1500 m, 15-16 Jul 1942, *J. A. Steyermark 48897* (F), Cerro Victoria, Sierra de los Cuchumatanes, near Barillas, 1800-2000 m, 29 Jul 1942, *J. A. Steyermark 49721* (F). Costa Rica. Prov. Alajuela, La Palma de San Ramón, 26 Sep 1924, *A. M. Brenes 1113* (NY), Upala, Bijagua El Pilón, subiendo por la margen derecha del Rio El Roble hasta el Volcán Tenorio, 1000-1400 m, 9 Jul 1988, *G. Herrera 2027* (MO), Upala, Bijagua El Pilón, ladera Atlántica del Volcán Tenorio, cuenca alta del Rio Celeste, 1500-1800 m, 23 Jul 1988, *G. Herrera 2144* (F, MO), San Ramón, Los Angeles. Reserva Biológica Alberto M. Brenes. Siguiendo la Fila Volcán Muerto hasta el Llano Costa Rica, 1300 m, 14 Oct 1994, *G. Herrera 7380 & H. Pérez Z.* (F), La Palma de San Ramón, 1250 m, 25 Oct 1924, *A. M. Brenes 2338* (F), La Palma, 1125 m, 29 Sep 1925, *A. M. Brenes 247(1434)* (F), Guanacaste, Parque Rincón de La Vieja. Liberia. Cabeceras de Quebrada Rancho Grande, bosque circundante a Meseta Aguacatales, 1350-1400 m, 2 Dec 1987, *G. Herrera 1484* (MO), Prov. Cartago, on road between Tapanti and the Tausito-Selva area, 1400-1500 m, 25 Aug 1975, *J. Utley, K. Utley 2998* (F), San Jose, 10 km NW of Guápiles, Limón 65 m, Aug 1982, *L. D. Gómez 18500* (MO), Prov. Cartago, forested knoll just W of Quebrada Casa Blanca, Tapantí, ca. 1350 m, 26 Dec 1984, *M. Grayum et al. 4652* (MO), forest on ridge between Quebrada Casa Blanca and road to Transito, Tapantí, ca. 1400 m, 10 Aug 1984, *M. Grayum, B. Jacobs 3722* (MO). Panama. Prov. Coclé, Caribbean side of divide et El Copé, 200-400 m, 3 Feb 1983, *C. Hamiton, G. Davidse 2625* (MO), summit at Alto Calvario, 7 km N of El Cope, 850 m, 19 May 1977, *J. P. Folsom, R. Button 3316* (MO), summit at Alto Calvario, low montane cloud forest, ridge-type vegetation, 900 m, 4 Apr 1977, *J. P. Folsom, R. Robinson 2407* (MO), El Valle de Anton, crest of Cerro Pajito, 1100 m, 28 Sep 1946, *P. H. Allen 3756* (MO), near Aserradero El Copé, north of El Copé, stream and hills east of sawmil, 750-800 m, 1-2 Nov 1980, *R. L. Dressler 5966* (FLAS), Pedregales, SSW of Rio Blanco del Norte, 22 Feb 1982, *R. L. Dressler 6030* (FLAS, MO), near summit of Cerro Gaital, N. of El Valle de Antón, 9 Jul 1982, *R. L. Dressler 6073* (FLAS), hills N of El Valle, E slope and ridges leading to Cerro Gaital, 900-1000 m, 27 Jun 1982, *S. Knapp 5778* (MO), Prov. Bocas del Toro, vicinity of Fortuna Dam. Along pipeline road leaving road to Chiriqui Grande at continental divide, 2.8 road-miles from divide, 850-950 m, 25 Jun 1986, *G. McPherson 9679* (MO), Prov. Chiriquí, vicinity of Fortuna Dam. Forest above lake, 1100 m, 6 Aug 1986, *G. McPherson 9847* (MO), Fortuna Dam Site, top of mountain above camo to south, 1700 m, 13 Sep 1977, *J. P. Folsom et al. 5409* (MO), camp Hornito, Fortuna dam site, 1200-1500 m, 16 Aug 1976, *R. L. Dressler 5391* (FLAS), Cerro Colorado. Bocas Road, 1500 m, 17-18 Feb 1977, *J. P. Folsom, L. Collins 1774* (MO), Prov. Veraguas, 6.4 km outside of Santa Fe on the road that passes the agriculture school, headed toward the cordillera, 5 May 1977, *J. P. Folsom 2954* (MO), Cerro Colorado, border of Chirigui and Bocas del Toro provinces, along intersection of Bocas Road with main ridge road, 11.8 km from Chami along path headed into Bocas del Toro, 1400-1700 m, 24 Oct 1977, *J. P. Folsom 6115* (MO), Prov. Darién, Ridge north of Cerro Pirre, 1050-1200 m, 12 Jul 1977, *R. L. Dressler 5663* (FLAS), Ridge north of Cerro Pirre, between Cerro Pirre top and Rancho Plastico, 1200-1400 m, 14 Nov 1977, *J. P. Folsom et al. 6300* (MO), Prov. Panama, area surrounding Rancho Chorro, mountains above Torti Arriba. Canazas mountain chain, 400-700 m, 1 Dec 1977, *J. P. Folsom et al. 6692* (MO), camino entre la cima maxima y la segunda cima. Hierba terrestre de 30 cm. Hojas verde oscura con líneas más claras, *J. Polanco 4018* (PMA), Prov. Veraguas, vicinity of Continental divide, between third branch of Rio Santa Maria and drop-off to lowlands, 650-750 m, 16-17 Nov 1974, *R. L. Dressler 4821* (FLAS), ridge east of Cerro Tute. NW of Santa Fe, 1000-1200 m, 30 Oct 1977, *R. L. Dressler 5734* (FLAS), Cerro Arizona, above Escuela Alto de Piedra, west of Snta Fe, 23 Oct 1980, *R. L. Dressler 5958* (FLAS). Colombia. Rio Vangolis on the highlands from Popayán, 1700-2000 m, Jan 1897, *F. C. Lehmann 10038* (K), Fusagasugá, 23 Dec 1852, *F. Holton s.n.* (K), Dept. Antioquia, Mun. Jardin, Microcuenca El Clavel, Reserva Natural Cuchilla Jardín Támesis, 2000-2400 m, 18 May 2006, *J. A. Pérez Zabala et al. 2619* (MEDEL), Dept. Valle del Cauca, Finca Zingara. Km 4 via a Dapa, corregimiento de la Elvira, cordillera Occidental, 1900 m, 12 Feb 1994, *J. Giraldo-Gensini 168* (MO), Mun. Yotoco, Hacienda Hato Viejo, 1875 m, 17 Feb 2010, *M. Kolanowska & O. Pérez s.n.* (COL, UGDA- drawing), San Antonio, TV tower, *D. L. Szlachetko 9158* (UGDA), Mun. Cali, KM 18 road Cali-Buenaventura, 2020 m, 16 Dec 2010, *M. Kolanowska 201* (COL, UGDA- drawing), Dept. Meta, Reserva Nacional de La Macarena. Pico Renjifo, 1300-1780 m, 29 Mar 1957, *J. M. Idrobo 2485* (COL), Dept. Cundinamarca, Santandercito, La Rambla, 2000 m, 2 Feb 1949, *M. Schneider 485* (COL, NY), Nevada St. Marta, *W. Purdie s.n.* (K), *Sine loc.*,23 Dec 1852, *F. Holton s.n.* (NY). Ecuador. Prov. Morona-Santiago,/Zamora-Chinchipe border, Cordillera del Cóndor. Cultivada por Ecuagenera, Cuenca, Ecuador, 1300 m, 24 Sep 2001, *A. Alvarez et al. 2917* (NY), Cordillera del Cóndor, Cuangos, 20 km east of Gualaquiza, near disputed Peru-Ecuador border, 1500 m, 19 Jul 1993, *A. Gentry 80242* (MO), Morona, Asociación Shuar Sevilla, Comunidad Angel Ruby, cima de la Cordillera junto al. camino Angel Ruby-Transcutucu, 2100 m, 23 Mar 2002, *L. Suin et al. 1188* (MO), Rio Chihuasi, 25 km SE of Logroño, Cordillera de Cutucú, 800-1000 m, 16 Jan 1976, *M. Madison, F. R. Coleman 2564* (MO), Prov. Zamora-Chinchipe, at the north slope of the Cordillera del Condor near Paquisha, 1450 m, 4 Feb 1987, *A. Hirtz 3115, C. Luer, J. Luer* (MO), Rio Jamboe, 12 km north of Zamora, 1000 m, 23 Nov 1988, *C. H. Dodson et al. 17842* (MO), Prov. Azuay, along road from Gualaceo to Chiquinda, 2600 m, Jan 1989, A. Hirtz et al. 4005 (MO), Prov. Santiago-Zamora, along Quebrada Honda, vicinity of Rancho Achupallas. Along river in narrow wooded valley with Cinchona "capuli", 2500-2700 m, 10 Oct 1943, *J. A. Steyermark 64559* (F), Prov. Napo, Archidona, Reserva Ecologica Antisana, Comunidad Shamato. Entrada por km 21-Shamato. Camino Sardinas-Shamato, 1700 m, 27 Apr 1998, *J. L. Clark et al. 5328* (MO), Archidona, Reserva de Biósfera Sumaco. Vertiente norte del Volcán Sumaco. Comunidad Pacto Sumaco, 1525 m, 18 Feb 2003, *W. Farfán 426* (MO), Archidona, Parque Nacional Sumaco-Galeras. Cumbre de la Cordillera de Galeras, 1690 m, 11 Mar 2003, *W. Farfán 523* (MO), Prov. Pastaza, shell-Mera rainforest, 2 km N of Shell-Mera, 1050 m, 6 Jun 1968, *L. Holm-Nielsen, S. Jeppesen 340* (F). Peru. Dept. Amazonas, ca 12-18 trail km E of La Peca in Serrania de Bagua, 1800-1950 m, 14 Jun 1978, *A. Gentry et al. 22923* (MO), Prov. Luya, Ocallí, anexo Ouispe, propiedad de San Estebau Fernández entraudo por "Galeras", 1920-2300 m, 19 Jun 1991, *C. Díaz et al. 4399* (MO), Prov. Condorcanqui. Cordiller del Condor, Puesto de Vigilancia Alfonso Ugarte, cabeceras del Rio Comainas, tributario al. oeste del Rio Cenepa. Subiendo cuchillo al. oeste del campamento, 1500-1700 m, 25 Jul 1994, *H. Beltran, R. Foster 1294* (F), Prov. Bagua, 12 km E of La Peca (by trail), 1700 m, 23 Jun 1978, *P. Barbour 2509* (MO, NY), Prov. Bagua, ca. 20 km by trail E of La Peca, 6560 ft., 20 Jul 1978, *P. Barbour 2733A* (MO), Dept. Cusco, Urubamba, Machu Picchu, above the second waterfall of the Rio Mandor, 3.7 km from Machu Picchu, 2220 m, 7 Jun 1982, *B. Peyton, S. T. Peyton 498* (MO), Dept. Pasco, prov. Oxapampa, Chontabamba, west of Oxapampa, low pass between Chontabamba and Suissa, 2100 m, 27 Dec 1983, *R. Foster, D.N. Smith 7573* (F). Bolivia. *Sine loc*., *M. B. Lectae 2908* (US), *Sine loc*., *M. B. Lectae s.n.* (NY). Guyana. Potaro-Siparuni Region, Mt. Ayanganna, east face, area near camp at base of second of four escarpments, 1120 m, 28 Jun 2001, *H. D. Clarke et al. 9606* (NY). Venezuela. Bolivar, Uaipan-tepui, the summit of the West Peak of Uaipan, 1980 m, 4 Mar 1967, *G. Agostini, T. Koyama 7488* (NY), forested west-facing slopes with sandstone boulders, between camp 2 and camp 3, northwestern part of Abácapa-tepui, 850-1100 m, 18 Apr 1953, *J. A. Steyermark 75114* (F), Carabobo, 22 Mar 1951, *H. Gines 4265* (US), Mérida, woods above Las Cuadras, along Quebrada Molino, north of Torondoy, 1820-2255 m, 27 Mar 1944, *J. A. Steyermark 55780* (F, NY), Territorio Federal Amazonas, Cerro Duida, southeastern-facing sandstone bluffs near Caño Negro (tributary of Caño Iguapo), 1095-1520 m, 26 Aug 1944, *J. A. Steyermark 58052* (F), Trujillo, municipio Boconó, Parque Nacional Guaramacal, on road from Boconó to Guaramacal, SE of Guaramacal, 2100 m, 15 Jul 1995*, L. J. Dorr et al. 8126* (MO, US), Territorio Federal Amazonas, Dept. Rio Negro, Cerro Aracamuni summit. Proa camp, 1400 m, 27 Oct 1987, *R. Liesner, G. Carnevali 22537* (MO). Brazil. São Paulo, Angatuba. Fazenda do Serviço Florestal, *M. Emmerich, R. Dressler 2841* (R), Rio de Janeiro, 22 Jan 1932, *A. Bade 11327* (R), Paraná, Quatro Barras. Terricola, flor creme eaverdeada, labelo levamente vinoso, 1100 m, 1 Dec 1967, *G. Hatschbach 15684* (F, US), Paraná, Brejatuba, Mun. Guaratuba, 3-5 m, 5 Feb 1987, *J. M. Silva s.n.* (US), Rio de Janeiro, Matto, vicinity of Macieiras, Mt. Itatiaya, Esração biologica, 1960 m, 9 Jan 1929, *L. B. Smith 1770* (US), São Paulo, Angatuba, Fazenda do Serviço Florestal, 23 Feb 1966, *M. Emmerich 2841, R. Dressler* (K), Paraná, Serra do Mar, Ypiranga in silvia primara ad terram, 15 Jan 1914, *P. Dusén 14461* (F, K, MO, NY), Paraná. Acarehy in silva primaeva and terran sphagnosam, 10 May 1915, *P. Dusén 17025* (MO), Marumbí, Paraná, 800 m, 13 Feb 1904, *P. Dusén s.n.* (R), *Sine loc*., *F. Sander & Co. s.n.* (K), Monte Alegre, 850 m, *P*. *Dusén 9022* (S).
